# Gout in the Common Fowl

**Published:** 1848-06

**Authors:** 


*Gout in the Common Fowl.*—During the painting of a hen-house, the birds (previously in perfect health) had been transferred for a few days to a damp cellar; where presently some of them sickened and rapidly died. One of the bodies was sent me by a friend, on the chance of its being a pathological curiosity; and such certainly it proved to be. The pericardium and peritoneum were stuffed with a white deposit; similar material lay in all the larger joints; and masses of it had thoroughly broken up the structure of both kidneys. On chemical analysis, I found it to consist entirely of the lithates of soda and lime; the latter predominating.—*Mr. Simon's Inaugural Address.*

